# Development of a mobile application for screening the risk of premarital sex among adolescents in Northern Thailand

**DOI:** 10.1186/s12889-026-27807-1

**Published:** 2026-05-21

**Authors:** Archin Songthap, Civilaiz Wanaratwichit, Sunsanee Mekrungrongwong

**Affiliations:** Department of Community Health, Faculty of Public Health, Naresuan University, 99 M.9, Thapho, Muang District, Phitsanulok, 65000 Thailand

**Keywords:** Premarital sex screening, Mobile application, Adolescents, Sexual behavior, Northern Thailand

## Abstract

**Background:**

Despite existing efforts, premarital sex among Thai adolescents remains a critical public health concern, exacerbated by nearly universal smartphone access and prolonged daily digital exposure. Traditional surveillance often fails to provide the real-time, private intervention needed in this digital era. This research and development (R&D) design aimed to develop a mobile application for screening the risk of premarital sex among adolescents in Northern Thailand.

**Methods:**

This research and development (R&D) study was conducted in three phases: (1) identifying 14 significant factors affecting premarital sex through a comprehensive literature review, (2) developing a mobile application via the Line Official Account platform, and (3) evaluating its diagnostic efficacy using a cross-sectional design. The sample consisted of 773 students (Grades 10–12 and years 1–3 vocational certificate) in Phitsanulok Province, Northern Thailand, recruited through stratified random sampling. Data collection was performed through real-time, self-administered digital assessments within the application. Statistical analyses focused on screening accuracy, including sensitivity, specificity, positive predictive value (PPV), negative predictive value (NPV), and the Area Under the Curve (AUC) of the Receiver Operating Characteristic (ROC).

**Results:**

The participants had a mean age of 16.5 years (SD = 1.29). The overall prevalence of premarital sexual experience was 31.3%. Premarital sexual experience was more prevalent among males (33.1%) than females (29.8%), indicating notable sex differences in adolescent risk behavior. The developed application demonstrated high diagnostic performance with a sensitivity of 86.4%, specificity of 70.0%, and an Area Under the Curve (AUC) of 87.9%. The Positive Predictive Value (PPV) and Negative Predictive Value (NPV) were 56.6% and 91.8%, respectively, indicating its effectiveness as a real-time screening tool for identifying at-risk adolescents.

**Conclusion:**

The 14-factor mobile application was successfully developed and validated, showing high sensitivity and accuracy in screening premarital sex risks among adolescents in Northern Thailand. This digital platform effectively bridges the gap in traditional surveillance by providing a private and real-time assessment tool. Consequently, it is recommended that schools and health providers utilize this application to facilitate early intervention and prevent the long-term consequences of risky sexual behavior.

## Background

Adolescence is the transition period between childhood and adulthood, characterized by significant physical, mental, emotional, and social changes [1]. During this stage, individuals develop a heightened interest in sexual matters as sexual development matures under the influence of hormones, leading to puberty [2]. Moreover, adolescents begin to experience attraction to others and develop sexual desires; however, their capacity for decision-making and emotional regulation is not yet fully developed.

In the digital age, adolescents can easily access various social media platforms and communicate extensively. This includes exposure to explicit content via the internet and mobile devices, as well as coordinating social gatherings at entertainment venues such as pubs and bars. Furthermore, these platforms facilitate peer invitations to consume alcohol and arrange meetings with the opposite sex. Such environments can heighten sexual arousal and lead to impulsive behavior, consequently increasing the likelihood of engaging in casual sexual activity [3].

Premarital sex during school age can cause health problems and academic performance, such as unwanted pregnancy, abortion, sexually transmitted infections and not being interested in studying [4]. The rate of unwanted pregnancy has continued to rise in many countries around the world. It is estimated that each year 21 million women aged 15–19 become pregnant in low- and middle-income countries. Approximately 21 million adolescent girls (aged 15–19) become pregnant annually in low- and middle-income countries, with nearly 50% of these pregnancies being unintended worldwide [5]. In the Philippines, it was found that teenagers give birth at a rate of 47 per 1,000 live babies [6]. Additionally, the abortion rate has increased in many countries, especially in Latin American countries and Sub-saharan Africa. Abortion rates among teenagers were found to be 41–107 and 49–145 per 1,000 women, respectively [7]. The period between 2020 and 2025 represents a critical transition in Thai adolescent behavior. Based on national surveillance data, there has been a notable convergence in sexual debut rates between genders. While male rates rose from 24.1% in 2020 to 35.0% in 2025, female rates saw a sharper increase from 16.9% to 28.2% during the same period. This trend, coupled with the decreasing average age of first intercourse (now at 15.0 years), highlights an urgent need for the mobile application [8]. The ‘digital-first’ approach of the application directly addresses this shift, providing a private screening tool that matches the 98.9% smartphone penetration among this demographic [9]. More alarmingly, the incidence of STIs among youth aged 15–24 has surged nearly threefold, rising from 102.5 per 100,000 in 2020 to 290.4 in 2025. This divergence indicates that while clinical contraception is more accessible, risky sexual behaviors and unsafe sex are becoming more prevalent in the post-pandemic digital era, necessitating real-time, private screening interventions [10].

In the specific context of Thailand, the digital landscape has shifted significantly. By early 2025, smartphone penetration among Thai high school and vocational students reached nearly 98.9% [11]. Recent data indicates that Thai adolescents (Gen Z) now spend an average of 7 hours and 54 minutes online daily, primarily through mobile devices [12–13]. Notably, LINE remains the most prevalent communication platform, used by over 85.7% of the internet-using population [14]. While this connectivity offers educational benefits, it also increases private and unmonitored access to suggestive content and online dating platforms, creating a ‘digital-first’ risk environment that influences sexual arousal and decision-making among youth. In Thailand, child protection and reproductive health are guided by the Act on Prevention and Solution of the Adolescent Pregnancy Problem [15]. However, local mechanisms primarily relying on teacher observations or infrequent paper-based surveys often fail due to social stigma and the ‘saving face’ culture, which discourages honest self-reporting. Adolescents frequently perceive these traditional methods as intrusive rather than supportive.

Based on previous studies, the primary factors influencing adolescent sexual intercourse include age, grade point average (GPA), smoking, having a partner, cohabitating with a partner, and alcohol consumption [16]. Yau et al. [17] found that self-efficacy in preventing sexual activity was significantly related to premarital sex. Furthermore, perceiving that peers had partners was associated with sexual involvement [18], as were curiosity and the desire to experiment [19]. Other significant contributors included a lack of parental supervision or living alone [20, 21], being in private with a partner [22], online pornography addiction [23], substance abuse [24], and attending vocational schools [9].

The literature reviews reveals that no mobile application has yet been developed specifically for screening the risk of premarital sex among adolescents. In Thailand, current technology is limited to a single application that assesses six broad aspects of student life, including academics, physical health, family, finances, substance abuse, safety, and general sexual behavior [25]. Similarly, in other countries, existing mobile applications primarily focus on providing general information regarding sexual and reproductive health rather than offering direct risk screening [26]. While traditional health surveillance exists, it often fails to capture real-time behavioral shifts in the digital era. There is an urgent need for a tool that bridges the gap between clinical screening and adolescent lifestyle. This study justifies the development of a mobile application as a critical intervention to provide immediate, private, and accessible risk assessment, which is vital for preventing long-term public health consequences like unintended pregnancies and STIs in a rapidly changing social landscape. Therefore, the primary objective of this study was to develop and evaluate the efficacy of a mobile application specifically designed for screening the risk of premarital sex among adolescents in Northern Thailand. Specifically, the study aimed to select and apply key risk factors from existing literature to develop the screening tool, design a digital screening platform via the LINE Official Account (Line OA), and evaluate its diagnostic performance in terms of sensitivity, specificity, and predictive values. For these reasons, we were interested in developing a mobile application for screening the risk of premarital sex among adolescents aged 15–19 years or grade 10–12 high school students and year 1–3 vocational certificate students. This application can be downloaded for use on mobile phones and can assess the risk immediately (real-time). The mobile application also provided basic advice on preventing the risk of premarital sex to students. This will help to reduce the risk of premarital sex and its consequences, such as risky sexual behavior, unwanted pregnancies, abortion, and sexually transmitted diseases in adolescents.

## Methods

### Study design, setting, and participants

This research and development (R&D) design was conducted and divided into 3 phases;

*Phase 1* was to study of factors affecting premarital sex among adolescents from the previous studies. The selected factors were used to determine the risk of premarital sex. The steps in this phase were as follows.

1. A comprehensive literature review was conducted to identify predictors of premarital sex. Out of 25 initially screened articles, 8 studies were selected based on the following inclusion criteria:
  1. Utilization of multiple logistic regression as the primary statistical analysis.
  2. Reporting of adjusted odds ratios (Adj OR) with 95% confidence intervals (95%CI).
  3. Statistical significance at *p* < 0.05.
  4. A study population specifically consisting of adolescents.
  5. Geographical and socio-cultural contexts relevant to Thailand (e.g., SE Asian and similar developing regions).
    While the significant factors varied across the 8 studies due to different regional dynamics, the selection process ensured that the variables shared similar controlled characteristics. The pooled Adj OR scores from these robust studies served as the quantitative baseline for categorizing factors into risk levels (low, medium, high). The selection of 8 peer-reviewed studies provided sufficient statistical power and a reliable evidence-based foundation for the subsequent development of the screening tool.
2. All selected factors were scored based on the adjusted odds ratio found in the previous studies. A scoring of factors was provided for the study participants to assess the risk of premarital sex in the mobile application. We applied the factor scoring guidelines of Johnson et al. [27] which consisted of 4 levels; low (AOR = 1.01–1.50), medium (AOR = 1.51-2.00), high (AOR = 2.01–3.99), and very high (AOR ≥ 4.00). The scores for low, medium, high and very high levels were 1, 2, 3, and 4, respectively. The reference group of the significant factors was given to be 0. The scoring weight for each of the 14 factors (Table 1) was assigned based on the strength of association (Adj OR) found in the literature, while the cumulative score thresholds were adopted from the Ministry of Public Health’s standardized criteria [28]. This hybrid approach combines recent empirical evidence with established public health monitoring standards, ensuring both the sensitivity of the tool and its alignment with national health policies. Their scoring weights and total risk thresholds (0–13 points for no risk, 14–27 points for medium risk, and 28–41 for high risk) were mapped to the Ministry’s standardized assessment protocol. The standard criteria classify risk levels based on total scores, categorized as low risk (0–30.00%), medium risk (30.01–69.99%), and high risk (70.00–100.00%). This ensures that our application remains consistent with the professional guidelines used by health educators and practitioners in Thailand.

*Phase 2* focused on the development of a mobile application designed to screen premarital sexual risk among adolescents. This application was integrated into the Line Official Account (Line OA) platform to ensure cross-platform compatibility with all smartphones. The development process was divided into two primary components:

- Content Development: This involved selecting key factors influencing premarital sex to construct the risk assessment tool. Furthermore, the application provided targeted prevention guidelines categorized into three risk levels: low, medium, and high.
- Interface Design and User Experience: This phase focused on technical components, including visual elements, color schemes, and typography, optimized for ease of use.

Content validity was rigorously evaluated by a panel of five experts specializing in health counseling, adolescent studies, social sciences, health behavior, and computer science. The application was assessed based on utility, feasibility, appropriateness, and accuracy. To quantify the results, the Index of Item-Objective Congruence (IOC) was employed. All items yielded IOC scores ranging from 0.60 to 1.00, indicating that the application met the established standards for validity.

*Phase 3* proposed to evaluate the efficacy of the mobile application for screening the risk of premarital sex among adolescents. In this phase, a cross-sectional study was conducted between January and June 2023.

### Participants and setting

A total of 5,610 population consisted of grades 10–12 secondary school students and year 1–3 vocational students (equivalence to grade 10–12) in Phitsanulok Province, Northern Thailand. The sample size was calculated from the formula for estimating proportions with a known population. We calculated the sample size based on a population of 5, 610, the prevalence of premarital sex among adolescents was 30% [9], the error estimation (e) was 0.03 and alpha (α) was 0.05. As a result, a total of 773 participants were in this phase. The inclusion criteria for the study participants included; (1) being students aged 15–19 years, (2) having a mobile phone, and (3) being able to participate in the research. The exclusion criteria were; (1) being ill on the data collection date, (2) being transferred to other areas, and (3) being unable to complete the data in the mobile application. We randomly selected 3 schools into the study by simple random sampling including 2 secondary schools out of 12 and 1 vocational school out of 6. Stratified random sampling was explored to select the study sample. Number of students in each school and class level were calculated using proportionate to size. We then randomly chose students from each class level among 3 schools by systematic random sampling.

### Research tool

The tool used in Phase 3 was a mobile application for screening the risk of premarital sex among adolescents which was composed of selected factors affecting premarital sex found in Phrase 1 to measure the risk levels of premarital sex. We added 1 more question in the application about history of having sex to confirm premarital sex of participants.

### Data collection

We visited each school to brief the directors and health teachers on the research objectives. Subsequently, appointments were made with selected students to explain the research goals, data collection methods, and ethical protocols. Participants consisted of students who provided their own assent and obtained parental consent. These students then assessed their risk of premarital sex via a mobile application, using a link sent through LINE. The assessment took approximately 10 min, after which the data were recorded into a software program for analysis.

### Data analysis

Information on risk factors for premarital sex was analyzed by frequency and percentage. The efficacy of the mobile application in screening for premarital sex risk was evaluated by comparing the risk classification of application against the reported history of sexual intercourse (the gold standard). The performance indices, including sensitivity, specificity, positive predictive value (PPV), negative predictive value (NPV), and the Area Under the Curve (AUC) of the Receiver Operating Characteristic (ROC), were calculated as detailed below:

The diagnostic test of the mobile application was evaluated by comparing the predicted risk levels (based on the 14-factor scoring) against the actual history of sexual intercourse (the gold standard). Sensitivity was measured as the proportion of participants with a history of sexual intercourse who were correctly identified as ‘at risk’ by the application. Specificity was the proportion of those without sexual experience who were correctly identified as ‘no risk’. Positive Predictive Value (PPV) and Negative Predictive Value (NPV) were calculated to determine the probability that the application’s risk classification correctly reflected the actual sexual history of participants. Additionally, the Receiver Operating Characteristic (ROC) curve was plotted to determine the optimal cutoff point, and the Area Under the Curve (AUC) was used to represent the overall diagnostic accuracy of the tool.

## Results

### Phase 1: factors affecting premarital sex among adolescents from a review of related previous studies and factor scoring

From the previous studies, fourteen factors affected premarital sex among adolescents in Northern Thailand including; (1) being male (AOR = 2.51, 95%CI = 1.42–3.85), (2) being aged 17 years and over (AOR = 2.33, 95% CI = 1.01–5.36, 3) studying in vocational school (AOR = 1.74, 95% CI = 1.21–3.66, 4) having a last semester GPA (grade point average) less than 3.00 (AOR = 1.54, 95% CI = 1.01–2.37), 5) receiving insufficient money for school (AOR = 5.98, 95% CI = 2.77–8.65), 6) alcohol drinking (AOR = 2.18, 95% CI = 1.34–4.97), 7) cigarette smoking (AOR = 2.55, 95% CI = 1.51–4.29), 8) drug use (AOR = 2.26, 95%CI = 1.11–4.61), 9) intending to have sex (AOR = 14.75, 95% CI = 8.61–25.27), 10) perceiving that friends have had sexเ (AOR 2.00, 95% CI = 1.21–3.75), 11 ) being unable to refuse having sex (AOR 1.41, 95% CI = 1.03–3.24), 12) having a boyfriend or girlfriend (AOR = 5.17, 95% CI = 1.71–15.57), 13) having the opportunity to be alone with his/her boyfriend or girlfriend (AOR = 6.40, 95% CI = 3.38–12.11), and 14) being addicted to pornographic media (AOR = 2.94, 95%, CI: 1.17–7.35). Then, we scored the 14 factors according to the adjusted odds ratio in the previous studies. Sum scores of all factors assessed by the study participants were used to categorize the risk levels of premarital sex (Table 1).

**Table 1:** Factors affecting the risk of premarital sex and factor scoring

Factors	Scores	Factors	Scores
1. Sex		8. Drug use	
Female	0	No	0
Male	3	Yes	3
2. Age		9. Intending to have sex	
< 17 years	0	No	0
≥ 17 years	3	Yes	4
3. Type of school		10. Perceiving that friends have had sex	
Secondary school	0	No	0
Vocational school	2	Yes	2
4. The last semester GPA		11. Unable to refuse having sex	
≥ 3.00	0	No	0
< 3,00	2	Yes	1
5. Receiving money for school		12. Having a boyfriend or girlfriend	
Sufficient	0	No	0
insufficient	4	Yes	4
6. Alcohol drinking		13. Having opportunity to be alone with his/her boyfriend or girlfriend	
No	0	No	0
Yes	3	Yes	4
7. Cigarette smoking		14. Being addicted to watch pornographic media	
No	0	No	0
Yes	3	Yes	3

Total: 41 scores

### Phase 2: development of a mobile application for screening the risk of premarital sex among adolescents

**Fig. 1:**
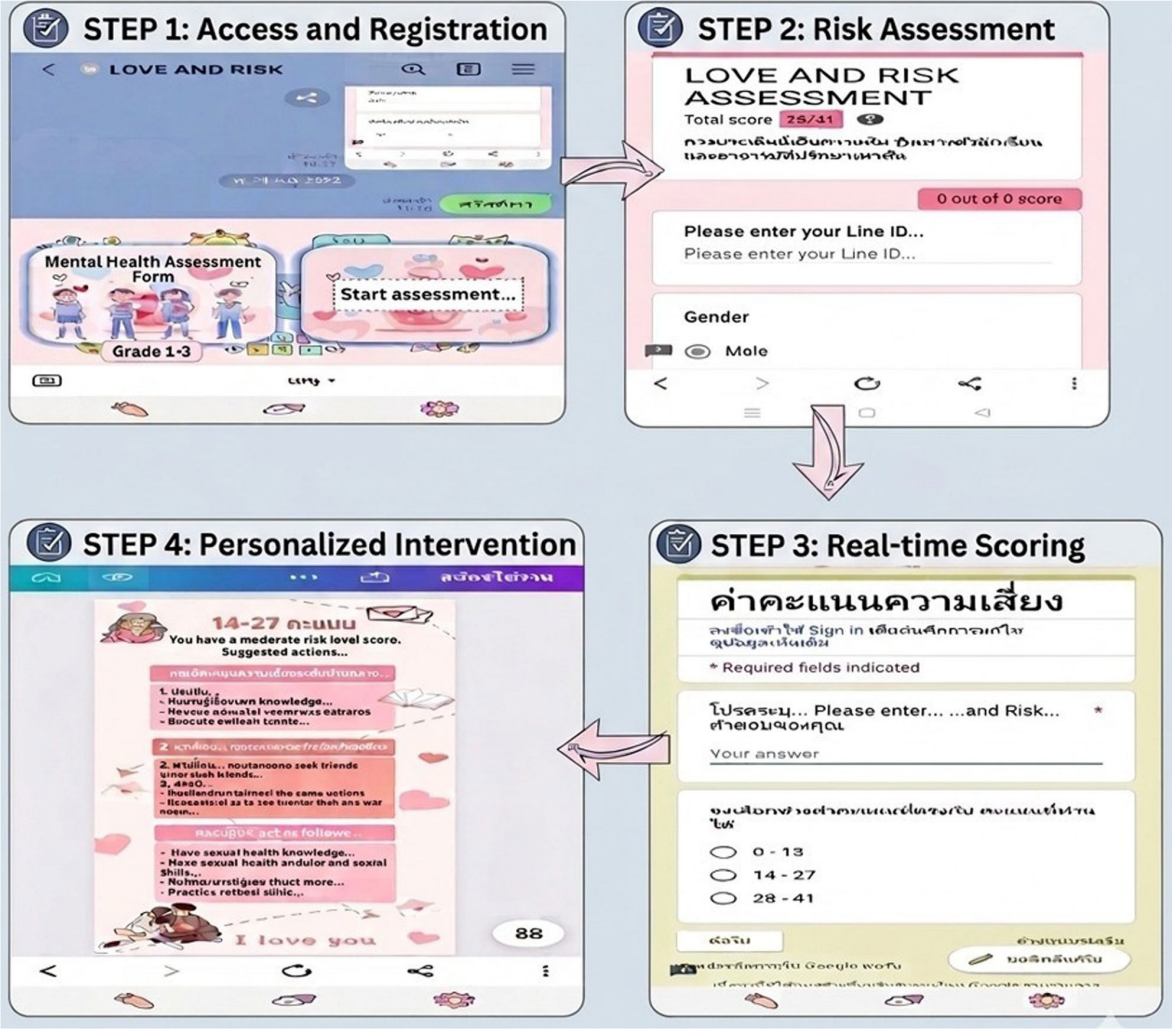
Screenshots of the mobile application interface (Thai version)

The application consisted of 2 sections; (1) In the content section, we put 14 factors affecting premarital sex among adolescents from Phase 1 on the application for risk assessment. (2) We developed the application’s components, including images, colors, text size and appropriate font style. The interface, layout, and operational flow of the developed application are visualized in Fig. 1. Figure 1 illustrates the key interface screenshots of application, detailing the operational flow from the welcome screen, the 14-factor risk assessment process, and the immediate, real-time result presentation with preliminary preventive advice.

The operational flow of the mobile application is structured into four sequential steps to ensure user-friendliness and privacy (Fig. 1):

- Step 1: Access and Registration: Users access the platform by scanning a uniquely generated QR code, which directs them to the Line Official Account for immediate registration.
- Step 2: Risk Assessment: Once logged in, users complete the 14-factor risk assessment via the ‘Assessment’ module to evaluate their individual risk profile.
- Step 3: Real-time Scoring: Upon completion, the application automatically calculates and displays the risk score, categorized into three levels: Low (0–13), Moderate (14–27), and High (28–41).
- Step 4: Personalized Intervention: Users can access tailored preventive guidance by clicking the ‘Risk Level’ link, which provides specific strategies and resources to prevent premature sexual engagement based on their screening results.

The content validity of the mobile application was evaluated by a panel of five experts in the fields of adolescent health, digital health, and behavioral sciences. The results of the Item-Objective Congruence (IOC) index assessment indicated that all items achieved an IOC score between 0.60 and 1.00, with an overall average of 0.85, confirming high content validity. These results are presented in Table 2.

**Table 2:** Results of content validity assessment of the mobile application using the Item-Objective Congruence (IOC) index

Component	IOC Range	Interpretation
Premarital sex risk factors	0.8-1.0	Congruent
Application operation	0.6-1.0	Congruent
Interface design	0.8-1.0	Congruent
Preventive guidance	1.0	Congruent
Overall Average IOC	**0.85**	**High validity**

Boldface indicates the overall average IOC assessment and its interpretation

### Phase 3: evaluating the efficacy of the application for screening the risk of premarital sex among adolescents

#### Premarital sex risk factors, history of having sex, and risk level of premarital sex

The study found that the majority of participants were female (54.2%) and aged 17 or older (50.8%). Most were secondary school students (84.2%) with a GPA of 3.00 or higher (60.9%), and 88.9% received an adequate daily allowance. Regarding risk behaviors, most respondents abstained from alcohol (56%), smoking (85.9%), and drug use (96.8%). While 53.7% did not have a partner, 63.4% of those who reported having opportunities to be alone together. Furthermore, 68.7% were virgins, 58.3% had no intention of having sex, and 88.0% felt capable of refusing sexual advances. Overall, 52.3% were classified as not being at risk of sexual involvement (Table 3).

**Table 3:** Number and percentage of classified by premarital sex risk factors, history of having sex, and risk level of premarital sex (*n* = 773)

Variables	*n* (%)
Premarital sex risk factors	
Sex	
Female	419 (54.2)
Male	354 (45.8)
Age	
< 17 years	380 (49.2)
≥ 17 years	393 (50.8)
Type of school	
Secondary school	651 (84.2)
Vocational school	122 (15.8)
The last semester GPA	
≥3.00	471 (60.9)
<3.00	302 (39.1)
Receiving money for school	
Sufficient	687 (88.9)
Insufficient	86 (11.1)
Alcohol drinking	
No	431 (55.8)
Yes	342 (44.2)
Cigarette smoking	
No	664 (85.9)
Yes	109 (14.1)
Drug use	
No	748 (96.8)
Yes	25 (3.2)
Intending to have sex	
No	451 (58.3)
Yes	322 (41.7)
Perceiving that friends have had sex	
No	274 (35.4)
Yes	499 (64.6)
Unable to refuse having sex	
Yes	680 (88.0)
No	93 (12.0)
Having a boyfriend or girlfriend	
No	415 (53.7)
Yes	358 (46.3)
Having opportunity to be alone with his/her boyfriend or girlfriend (*n* = 358)	
No	131 (36.6)
Yes	/227 (63.4)
Loving to watch pornographic media	
No	482 (62.4)
Yes	291 (37.6)
History of having sex	
Yes	242 (31.3)
No	531 (68.7)
Risk levels of premarital sex	
No risk	404 (52.3)
Have risk	369 (47.7)

#### Efficiency of the application for screening the risk of premarital sex among adolescents

The cut-off points derived from the Ministry of Public Health’s criteria were consistent with those generated by the software. To categorize the risk of premarital sex, the optimal cut-off scores were established at 0–13 for the ‘no-risk’ group and 14–41 for the ‘at-risk’ group. The performance of the mobile application was evaluated using the data from Table 4, which revealed a sensitivity of 86.4%, a specificity of 70.0%, a positive predictive value (PPV) of 56.6%, and a negative predictive value (NPV) of 91.8%.

**Table 4:** Number of with the cut points of 0-13 scores for the no-risk group and 14-41 scores for the risk group against history of having sex

Risk levels of marital sex	Having sex		Total
	Yes	No	
Risk group (0–13)	209	160	369
No-risk group (14–41)	33	371	404
Total	242	531	773

**Fig. 2:**
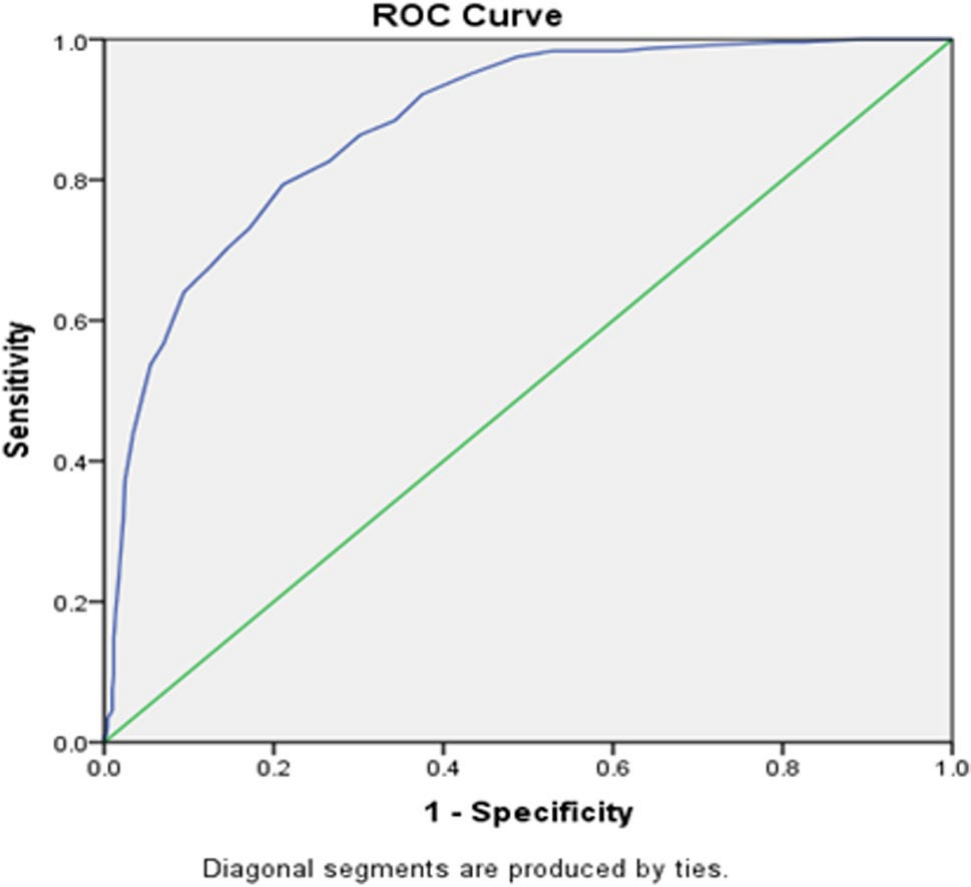
Receiver Operating Characteristic (ROC) curve for the adolescent premarital sex risk screening application

To validate the diagnostic efficacy of the 14-factor mobile application, a Receiver Operating Characteristic (ROC) curve was plotted (Fig. 2). The analysis yielded an Area Under the Curve (AUC) of 0.879 indicating high overall accuracy in discriminating between adolescents with and without premarital sexual experience. As demonstrated by the ROC curve analysis in Fig. 2, the optimal cutoff point for the application was determined to be 13 points. This empirical value effectively discriminates the ‘risk’ group from the ‘no-risk’ group and is perfectly consistent with the criteria established by the Health Education Division, Ministry of Public Health [27]. At this threshold, the application achieved a high sensitivity of 86.4% and an overall accuracy (AUC) of 87.9%, confirming its robustness as a screening tool.

Table 5 presents the predictive performance of the mobile application for screening premarital sex risk, stratified by sex. The analysis revealed distinct performance patterns between male and female participants. The application demonstrated higher sensitivity in males (94.0%) compared to females (79.2%), suggesting a superior ability to identify at-risk cases among male adolescents. Conversely, the specificity was significantly higher in females (85.0%) than in males (51.1%), indicating that the tool is more effective at correctly identifying non-risk individuals in the female group. The Negative Predictive Value (NPV) remained high across both groups (94.5% for males and 90.6% for females), showing that a “low risk” result from the application is highly reliable for both sexes. The Positive Predictive Value (PPV) was higher in females (69.2%) than in males (48.7%). Furthermore, the Area Under the ROC Curve (AUC) indicated high overall accuracy for both groups, with values of 88.0% for males and 91.4% for females. These results signify that the application possesses a “very good” to “excellent” discriminative ability to distinguish between individuals at risk and those not at risk of premarital sex in both genders.

**Table 5:** Predictive performance of the application stratified by sex (*n* = 773)

Performance Metric	Total sample (*n* = 773)	Male (*n* = 354)	Female (*n* = 419)
Prevalence of sex (%)	31.3	33.1	29.8
Sensitivity (%)	86.4	94.0	79.2
Specificity (%)	70.0	51.1	85.0
PPV (%)	56.6	48.7	69.2
NPV (%)	91.8	94.5	90.6
AUC of ROC (%)	87.9	88.0	91.4

## Discussion

In this study, the prevalence of premarital sexual intercourse among adolescents was slightly higher than that reported in a previous study of adolescents in Northern Thailand [16]. Our findings also showed a considerably higher prevalence compared to studies conducted among high school students both in Thailand [29, 30] and Ethiopia [31]. This discrepancy is primarily attributed to the inclusion of vocational certificate students in our sample, who demonstrated a substantially higher prevalence of sexual activity compared to secondary school students. This trend is consistent with the findings of Chintu et al., who noted that vocational students often experience greater residential autonomy and more frequent unmonitored opportunities with partners [32–34]. These environmental factors, combined with potentially less rigid school-based support systems, underscore the need for targeted digital screening in vocational settings.

The application’s efficacy is rooted in the integration of 14 key evidence-based variables, prioritized from Phase 1, which encompass individual behaviors (e.g., substance use, sexual intention) and socio-environmental factors (e.g., peer influence, situational opportunities). This structured approach aligns with the design philosophy of the Mental Health Checkup application by the Department of Mental Health, Thailand [35], which utilizes validated clinical indicators to screen for psychiatric risks. However, while the Mental Health app focuses on internal psychological states, our application expands this framework by incorporating situational triggers such as being alone with a partner which are specific to the Thai adolescent context. By prioritizing these high-odds ratio variables, our tool provides a more context-specific screening than generalized health platforms, allowing for immediate risk categorization and personalized intervention, much like the real-time mental health triage system used during the COVID-19 pandemic.

Social and situational factors also played a pivotal role. The perception of peer sexual behavior creates a perceived social norm that can diminish the perceived risk of premarital sex. When adolescents believe their peers are sexually active, they may feel social pressure to conform to avoid being “out of step” with their group [36]. This social influence is often compounded by physical opportunity. Having a boyfriend or girlfriend and the opportunity to be alone together, especially in private settings like rented rooms or during unmonitored after-school hours significantly lowers the threshold for sexual engagement. In these private moments, refusal self-efficacy is often tested, making situational control a critical factor in risk screening [37]. By incorporating these multifaceted factors, the developed application provides a holistic assessment that transcends simple demographic screening.

Our study showed that the best cutoff point generated by the ROC curve is at the 13-point level separated the no-risk group from the risk groups. This was the same value as the cutoff point that we established according to the criteria of the Health Education Division, Department of Health Service Support, Ministry of Public Health [28]. At this cut point, we found a sensitivity of 86.4%, a specificity of 70%, a positive predictive value of 56.6%, a negative predictive value was 91.8% and an Area Under the Curve (AUC) or the accuracy of the application was 87.9%. Since no prior research has evaluated a mobile application specifically for premarital sex risk screening, the efficacy of our tool was compared with screening instruments for other adolescent health behaviors. The sensitivity of our application was comparable to tools used for stress assessment [38] and slightly lower than those developed for depression [39], suicide risk [40], and pediatric psychosis screening [41]. Similarly, the specificity and overall accuracy of our tool were within a similar range to these established clinical instruments. These variations in predictive performance likely stem from differences in the study populations, the nature of the behaviors being screened, and the specific cut-off points utilized in each study. It can be seen that the efficiency of each type of screening tool is different. This might depend on the study samples, diseases, behaviors, and the cut point under the study. The predictive performance of the mobile application for screening premarital sex risk, stratified by sex indicated that although the application showed high overall accuracy for both genders, the lower specificity in males suggests a higher rate of false positives in this group. This may be attributed to the 14 identified risk factors being highly prevalent among male adolescents, even those who have not yet engaged in premarital sex.

In our opinion, the high sensitivity (86.4%) observed is the most critical achievement of this mobile application. In the context of premarital sex screening, missing a high-risk individual (False Negative) is far more detrimental than a false alarm. A high sensitivity ensures that we can capture the majority of at-risk students and offer them preventive education or counseling, which is much more cost-effective than addressing unintended pregnancies or STI outbreaks later. While the positive predictive value (PPV) was moderate at 56.6%, this is expected in screening tools used within populations where the prevalence of the condition. In this case, premarital sex (31.3%) is not predominant. Moreover, the high negative predictive value (NPV) of 91.8% underscores the application’s reliability in correctly identifying those who are truly at low risk, allowing health providers to focus their limited resources more effectively on the identified high-risk group. The visual representation of the ROC curve confirms the robust validation of our digital tool. An AUC of nearly 0.88 suggests that the application has an 88% chance of correctly distinguishing a high-risk adolescent from a low-risk one. This level of performance is superior to many traditional paper-based screenings and justifies the application’s use as a reliable real-time surveillance tool in school settings.

From our perspective, the transition to a mobile-based platform like Line OA is a strategic response to the cultural barriers in Thailand. Adolescents often feel stigmatized when discussing sexual health in person. We believe that the ‘digital distance’ provided by the application creates a safe space, encouraging more honest self-reports and reducing social desirability bias, which has traditionally hindered the accuracy of sexual risk assessments.

### Strengths and limitations

This study has several strengths. It introduces a novel digital screening tool for premarital sex risk among Thai adolescents via the Line Official Account platform, offering convenient, real-time, and cost-free access. The application was developed using an evidence-based R&D process and validated by multidisciplinary experts, demonstrating high sensitivity and overall accuracy. However, some limitations should be noted. Data were self-reported and may be subject to social desirability bias. The sample was limited to one province, which may affect generalizability. In addition, the cross-sectional design limits the ability to assess long-term predictive validity, and the lower specificity observed among male participants suggests the need for further refinement of the screening model.

The findings of this study have significant policy implications for adolescent sexual health management in Thailand. First, the high prevalence of sexual activity and risk factors among vocational students suggests that the Ministry of Education and the Office of the Vocational Education Commission should prioritize the integration of digital screening tools into routine student health surveillance systems. Transitioning from traditional paper-based assessments to a standardized mobile platform like the one developed in this study can provide real-time, large-scale data to inform regional health resource allocation. Second, the Ministry of Public Health could adopt this digital screening model as a primary preventive strategy within school health clinics (Secondary Care), enabling school nurses and counselors to identify high-risk individuals early and provide targeted interventions. This shift toward digital-first preventive policy not only reduces the social stigma associated with seeking sexual health advice but also offers a cost-effective solution to mitigate long-term public health challenges, such as unintended adolescent pregnancies and the spread of sexually transmitted infections (STIs) in the region.

### Conclusion and recommendations

This study successfully developed and validated a mobile application for screening premarital sex risk among adolescents in Northern Thailand. The application integrates multiple evidence-based risk factors into a user-friendly digital platform and demonstrates strong capability in identifying at-risk individuals while reliably distinguishing those at low risk. Overall, the findings support its potential as an accessible and practical tool for early screening and intervention in adolescent sexual health.

Based on these findings, future research should focus on longitudinal studies to evaluate the long-term predictive validity of the application and its impact on behavioral outcomes. Further refinement of the screening model is also recommended, particularly to improve specificity among male users. In addition, expanding and adapting the application to different cultural and regional contexts in Thailand would enhance its broader applicability.

## Data Availability

The data that support the findings of this study are available from Naresuan University Research Ethics Committee but restrictions apply to the availability of these data, which were used under license for the current study, and so are not publicly available. Data are however available from the authors upon reasonable request and with permission of Naresuan University Research Ethics Committee. Data is provided within the manuscript or supplementary information files.
